# Higher Landing Accuracy in Expert Pilots is Associated with Lower Activity in the Caudate Nucleus

**DOI:** 10.1371/journal.pone.0112607

**Published:** 2014-11-26

**Authors:** Maheen M. Adamson, Joy L. Taylor, Daniel Heraldez, Allen Khorasani, Art Noda, Beatriz Hernandez, Jerome A. Yesavage

**Affiliations:** 1 Department of Veterans Affairs, WRIISC and Sierra-Pacific MIRECC, Palo Alto, California, 94304, United States of America; 2 Department of Psychiatry & Behavioral Sciences, Stanford School of Medicine, Stanford, California, 94305, United States of America; University Medical Center Goettingen, Germany

## Abstract

The most common lethal accidents in General Aviation are caused by improperly executed landing approaches in which a pilot descends below the minimum safe altitude without proper visual references. To understand how expertise might reduce such erroneous decision-making, we examined relevant neural processes in pilots performing a simulated landing approach inside a functional MRI scanner. Pilots (aged 20–66) were asked to “fly” a series of simulated “cockpit view” instrument landing scenarios in an MRI scanner. The scenarios were either high risk (heavy fog–legally unsafe to land) or low risk (medium fog–legally safe to land). Pilots with one of two levels of expertise participated: Moderate Expertise (Instrument Flight Rules pilots, *n* = 8) or High Expertise (Certified Instrument Flight Instructors or Air-Transport Pilots, *n* = 12). High Expertise pilots were more accurate than Moderate Expertise pilots in making a “land” versus “do not land” decision (CFII: *d*′ = 3.62±2.52; IFR: *d*′ = 0.98±1.04; *p*<.01). Brain activity in bilateral caudate nucleus was examined for main effects of expertise during a “land” versus “do not land” decision with the no-decision control condition modeled as baseline. In making landing decisions, High Expertise pilots showed lower activation in the bilateral caudate nucleus (0.97±0.80) compared to Moderate Expertise pilots (1.91±1.16) (*p*<.05). These findings provide evidence for increased “neural efficiency” in High Expertise pilots relative to Moderate Expertise pilots. During an instrument approach the pilot is engaged in detailed examination of flight instruments while monitoring certain visual references for making landing decisions. The caudate nucleus regulates saccade eye control of gaze, the brain area where the “expertise” effect was observed. These data provide evidence that performing “real world” aviation tasks in an fMRI provide objective data regarding the relative expertise of pilots and brain regions involved in it.

## Introduction

Everyday decisions are often made in the presence of risk and uncertainty. Risk refers to multiple possible outcomes, positive and negative, that could occur with well-defined or estimable probabilities [1]. The neural correlates of financial decision making have been extensively investigated in the field of neuroeconomics where ‘risk’ refers to monitoring monetary outcomes. These studies suggest that decision making is a complex process where monetary gains and losses are associated with activity in multiple brain regions, namely, the basal ganglia, ventral prefrontal, insular and cingulate cortices [2]. Individual differences, such as age, brain injury, addiction, or past experience exist in the preference of risk and outcomes.

Naturalistic decision making, for instance in a real-world task, involves choices that can physically harm oneself or others, and may have delayed consequences [3]. In these situations, individual differences are key factors that shape brain activation and the decision making process. Few studies have characterized the decision making process that takes place in the real world – possibly due to the lack of ecologically valid tasks required to observe real-world behaviors [4]–[6]. In this study, we assessed naturalistic decision making and related brain function in a pilot's decision to land in bad weather—a decision making task that has obvious real-world consequences.

Some of the deadliest aviation accidents, with over 70% lethality, have taken place in the areas of flight planning and decision-making in bad weather [7]. This is because approach and landing are two of the most demanding and critical flight phases requiring formalized sequences of actions and judgments (e.g. perform a visual inspection for landing, lower the landing gear, extend the flaps, etc; see [8]). Navigating an aircraft is a complex, time-pressured activity, influenced by individual difference factors such as level of expertise, age, cognitive and neural capabilities [9]–[12]. For instance, higher expertise was associated with less deviation in lateral positioning during instrument approaches [13] and visual approaches [11], indicating experts' closer performance to the ideal landing approach pattern.

To elucidate the neural correlates of pilots' crucial decisions in real-world flying conditions such as bad weather, our study employed a series of short simulated instrument approach-and-landing scenarios in the fMRI scanner. The scenarios were based on those used in the Kennedy et al. [13] instrument landing-decision study, and were adapted to the fMRI environment. For the fMRI landing decision task, both the flight simulator's “thru the window” display and the instruments (which all airplanes have for flying without visual references) were modified for laptop display and projected on to the fMRI head coil mirror. An fMRI-compatible joystick was used for “flying” inside the scanner. The fMRI task was comprised of two scenarios: high risk (poor visibility due to heavy fog–legally unsafe to land) and low risk (better visibility due to medium fog–legally safe to land). During an instrument approach, pilots' eye movements are directed at the instruments, as well as the landing visual markers at the decision height (DH) of 200 feet altitude. No feedback or compensation was offered to study participants; the pilot's motivation to make a safe landing decision was personal safety and following the FAA rules for visual cues that need to come into view at the DH.

The characteristics of our real-world task led us to focus on certain brain regions involved in decision making. Traditionally, evidence from neuropsychological tasks such as the Iowa Gambling Task (IGT) [14] and the Balloon Analog Risk Task (BART) [15] have led to the development of a neuroscience systems-based model [16] identifying striatum (i.e. caudate and putamen), ventral medial prefrontal cortex (vmPFC), and amygdala as the brain regions involved in assessing the cost and benefit of a pending decision [17]–[18]. The caudate and putamen, as part of the basal ganglia, have a key role in contextually-based motor decision-making, i.e. deciding if and when to correct a given movement by initiating corrective sub-movements [19]. Moreover, the caudate, as part of the basal ganglia-superior colliculus (SC) pathway, plays a role in the initiation of saccadic eye movements [20]–[21]. Importantly, eye movement strategies have been associated with skill and expertise in baseball batting [22], billiards [23] and flying [24]. The involvement of eye motor movements in our aviation decision-making task and its role, in general, in skill and expertise persuaded us to choose bilateral caudate as the primary region of interest (ROI).

The present study used a comprehensive and relatively unbiased data-driven approach to create the primary ROI by combining large-scale meta-analysis of functional activation during decision making with structural ROIs from a human brain atlas. Specifically, we relied on the “Neurosynth” database (http://neurosynth.org) that contains automatically generated meta-analysis maps for several thousand psychological terms and topics [25]–[27]. Therefore, it is possible to compute whole-brain functional maps for individual cognitive and psychological concepts.

In this study we aimed to characterize, in terms of performance and brain activity, the individual contributions of aviation expertise to the crucial decisions pilots make when they land in bad weather. To do this, participating pilots varied in their aviation expertise.

## Materials and Methods

### Participants

There were 20 pilots who completed the study, ranging from 20 to 66 years of age (mean  = 48.4±13.5), with two levels of aviation expertise. Moderate expertise was defined as having a rating of IFR (Instrument Rated, allowing a pilot to fly in poorer visibility conditions using navigational instruments). High expertise was defined as having a rating of CFII (Certified Flight Instrument Instructor of IFR students) or ATP (eligible to fly Air-Transport planes). To be eligible to participate in the study, all pilots needed to have a current FAA Medical Certificate (Class III or higher), which assesses pilots' vision, hearing, and physical and mental health. Pilots' medical certificates were verified; they also had to be currently flying with at least 500 hours of total flight time. No special neurological, visual acuity, sleep disorder or hearing tests were performed. These pilots were recruited from the ongoing longitudinal Stanford/VA Aviation Study and surrounding aviation community including local airports and pilot gatherings. Eight had the basic “IFR' rating (Moderate expertise), and 12 were “IFR experts” in that they held CFII/ATP licenses and taught IFR procedures (High expertise). Mean years of education was 16.1 yr (*SD* = 1.9) with a range of 12–19 years. Mean total flight hours was 2399 h (*SD* = 1944 h, range: 368–10,000 h), and mean number of flight hours in the month prior to study participation was 22.2 h (*SD* = 27.8 h, range: 0–80 h). Of the 20 pilots, 18 were male, and 17 were Caucasian and non-Hispanic. Two additional pilots did not have complete data: one due to claustrophobia in the fMRI scanner and one due to movement during the scan that was greater than 4 mm. Table 1 describes demographic and cognitive ability characteristics of the pilots.

**Table 1:** Demographic characteristics (mean ±SD) by aviation expertise; Mean (SD). (High Expertise  =  CFII/ATP; Moderate Expertise  = IFR).

	IFR	CFII/ATP
	*n* = 8	*n* = 12
Age, y, mean ±SD*	55.1±16.4 (age-range = 20.5–66.1)	41.7±10.5 (age-range = 27.1–57.3)
Education, y, mean ±SD	16.9±2.1	15.5±1.6
Number White, non- Hispanic	7	10
Number Men	8	10
Total flight time, h, median ±SD*	1136±626	3662±3262
Past month flight time, h, mean ±SD*	2.6±2.0	33.7±29
Status of FAA Medical Certificate (Current)	8	12
Employment Status (Fulltime/Part-time/Retired/Self-Employed/Unemployed)	3/3/2/0/0	7/0/0/4/1
Self-report of regular exercise (Yes/No)	7/1	8/4
Health Problems (BP/High Cholesterol/Diabetes/None)	2/0/1/6	0/2/0/10

*p<.05, Satterthwaite, 12 df.

#### Ethics Statement

The study was approved by the Stanford University Institutional Review Board (IRB). All pilots gave written informed consent to participate, with the right to withdraw at any time.

### Equipment

**Figure 1:**
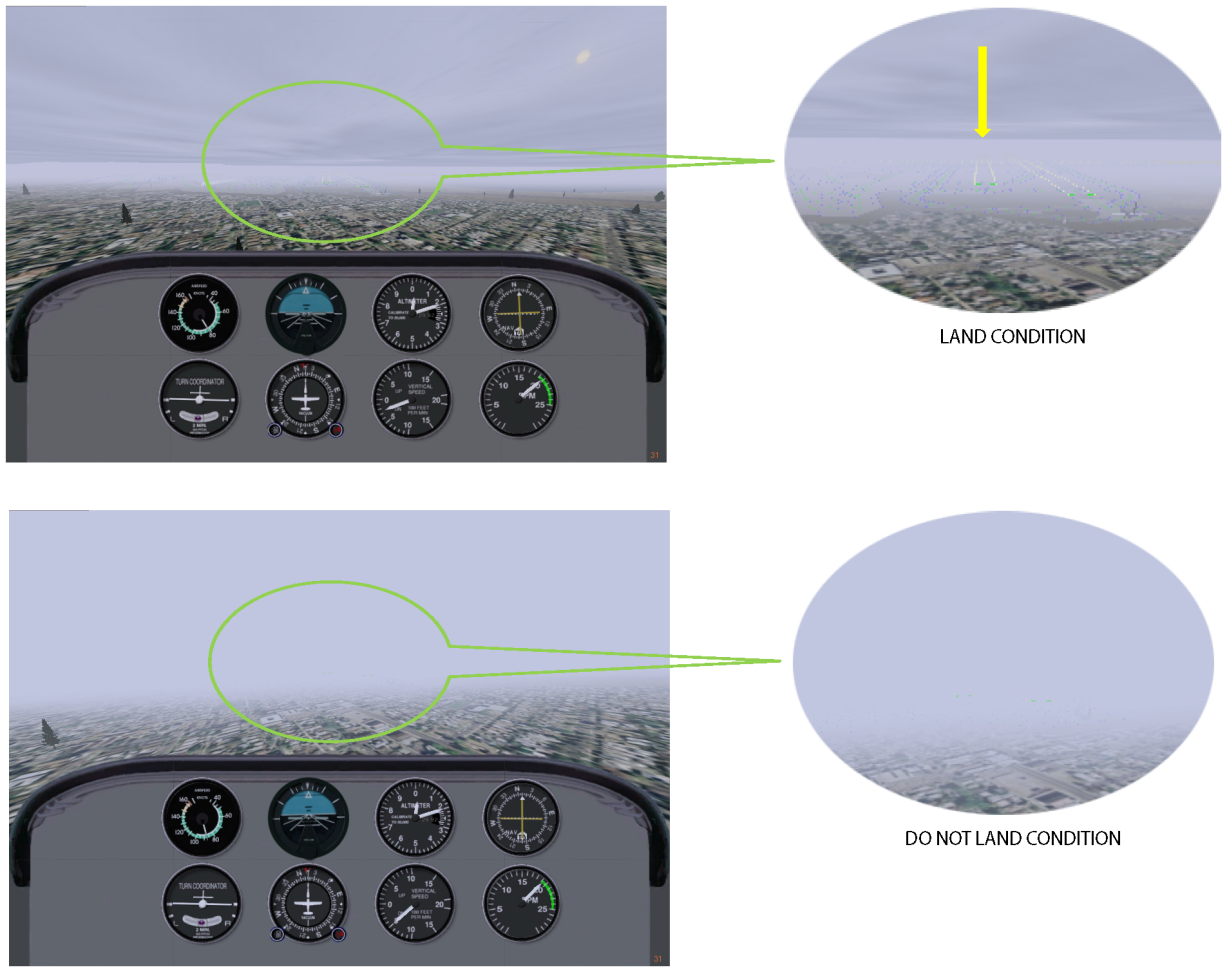
Landing Decision Task. Land Condition  =  Low fog levels and landing strip visible (yellow arrow); Do Not Land Condition  =  Heavy fog levels and landing strip is not visible.

The landing decision task was a simulation based on Kennedy et al., (2010) with design consultation from IFR & CFII/ATP pilots who worked in the laboratory. The simulation for a Cessna 172p was made using a modified version of FlightGear, an OpenSource simulator. The C/C++ programming ran on a high-end Nvidia and ATI GPU's under LINUX operating system. The flight dynamics model (FDM) was based on SimGear, an open source flight physics engine. All scenery and environment was rendered through OpenSceneGraph. A modified version of the "sacred six" (six essential cockpit instruments) along with the Instrument Landing System (ILS) receiver and an engine RPM gauge was rendered on the screen (see Figure 1). A scene modeling the San Francisco international airport (SFO) was rendered in the same window above the eight instruments. While viewing this scene, the pilot made an instrument (i.e., ILS) approach to the runway using an fMRI-compatible joystick. At each frame the pilot's inputs were sent to SimGear which simulated a single engine Cessna aircraft. All ILS signals were simulated in the software. The simulation on the laptop was shown via a projection system onto a mirror inside the fMRI scanner.

### Task Procedures

Pilots signed the consent form and provided demographic information including FAA certificates for verification. The task was first presented to the pilot on the laptop outside the scanner with the fMRI-compatible joystick. A research assistant, who was a CFII pilot, read the following preflight instructions to pilots: “You will fly multiple ILS approaches in this task. To do this, you will fly your ILS to a touch and go or a missed approach and then you will be taken to the next trial. You will continue to do several of these approaches. In trials that have green letters on the top of the screen, *fly an ILS approach but do not look up to* make a landing decision at 200 feet.” Next, pilots “flew” ILS approaches on the laptop using the joystick for 15 min in a practice session to become more familiar with the task and the joystick. They were then escorted to the scanner room where they first had additional practice inside the MRI scanner with the same scenarios and the joystick during the localizer and the anatomical scan. They then completed the task lying down in the scanner for actual data collection.

Each trial was approximately a 30 second approach scenario in a Cessna 172p with the DH to be made between 18–20 seconds into the approach (the FAA ‘DH’ of 200 feet). Each experimental trial started at 350 ft (∼a mile away from the runway) at 90 knots established on the glide slope. In actual flying an approach requires more time and ‘piloting’ several aircraft controls. In this ‘fMRI-compatible’ task, the flaps, throttle and the elevator trim of the aircraft were pre-set for approximately 500 ft/min descent at the beginning of each trial. Thus, to stay on course during the descent, the pilot primarily needed to control the ailerons by moving the joystick left or right. Because the elevator trim was pre-set, minimal forward and aft stick movements were needed to make pitch (elevator) corrections. The trim was set the same way for all trials.

On landing-decision trials, the pilot was to conduct an ILS approach on runway 1L at SFO, with different high visibility and low visibility conditions and make a decision whether to land or not. Six variations of fog density were presented during the landing decision task scenario, in which pilots flew multiple ILS approaches to the runway, each time making a “land/do not land” decision upon reaching DH altitude of 200 ft (∼70 m) above ground. In the three variations of fog density, although fog is present, the runway is visible at the DH, and thus should lead to the decision to land (referred to as “land” trials). In the other three variations, the fog is too thick to see the runway or other Federal Air Regulation Section 91.175 legally acceptable markers at the DH. Such a low level of visibility should lead to the decision to not land (“do not land” trials). The pilot flew an ILS approach until the plane descended to DH. On “land trials”, the correct response was to move the joystick forward to descend as one would move an aircraft yoke control. On “do not land trials”, the correct response was to move the joystick backward to climb.

Control trials were randomly intermixed with decision trials. To indicate a control trial, the instructions “fly an ILS approach” appeared on the top part of the screen in green letters. During the practice session the pilot had been instructed to *fly an ILS approach but not to look up to* make a decision at 200 ft. When the pilot flew an ILS control trial, they again primarily used left and right joystick movements to stay on course, but they continued their descent below 200 ft. This was done to maintain the need for motor control of the joystick *without* the need to make a decision whether or not to land. Once the pilot reached 150 ft, the aircraft was automatically reset at 350 ft to begin the next ILS trial. Then the pilot would either fly another ILS control trial; or, the instructions to not make a decision were cleared from the screen and the pilot would begin an ILS land/do not land decision trial. A total of 32 landing-decision approaches (16 "land" and 16 "do not land") and approximately 8–10 control trials were randomly presented during one 20–24 min long scan session. Note, there was no fixation in the experimental design, so there was no “rest” condition. The main reason for having a control condition similar to the task conditions was to have a tight contrast without the need for a fixation.

### Flight Gear Data Reduction and Statistical Analysis

Accuracy and reaction time at the DH (when pilot makes a decision to land or not to land) were calculated for each fMRI trial. The main behavioral outcome measure for the “land” versus “do not land” decision was the measure of sensitivity d′ (hits minus false alarms). A false alarm was scored when the pilot made an incorrect decision to land during a “do not land” trial (i.e. the fog was too thick to see the runway or other markers at the DH). A hit was scored when the pilot made a correct decision to land during a “land trial” (i.e. the fog was less dense). Correct rejections (not landing in a “do not land” trial) and misses (not landing in a land trial) were also tabulated. To examine the expertise effect, a t-test was performed on d′.

#### Approach variables (lateral deviations, aileron and elevator movements)

Small lateral deviations indicate the ability to maintain close alignment with the runway centerline. Ailerons are used to bank the aircraft and turn left or right and maintain the extended runway centerline while descending. Lateral deviations from the extended centerline are considered to be the primary approach measure, with size of movements in ailerons and elevators as secondary measures. (Note: elevator trim of the aircraft is preset at the start of the simulation). For all approach measures, positive scores indicate greater deviations from the ideal and thus worse flight control. As would be expected, Spearman's rank correlation indicated that ailerons was significantly correlated with lateral deviations (*r* = 0.86, *p*<.001).

### Image Acquisition, Processing and Statistical Analysis

#### FMRI data acquisition

Whole-brain imaging data were acquired on a 3 Tesla MRI system (General Electric Medical Systems) at the Lucas Imaging Center at Stanford University School of Medicine. The scan session included the following sequences: a) Three-dimensional gradient echo MRI (3D magnetization-prepared and rapid gradient-echo (MP RAGE)) of entire brain, TR (repeat time)/TE (echo time)/TI (longitudinal relaxation time)  = 10/4/300 ms (TR/TE  = 9/2 ms for SPGR), 15o flip angle, perpendicular to the long axis of the hippocampi as seen on sagittal scouts 0.976×0.976 mm2 in-plane resolution, 1.5 mm slab thickness, no gap (acquisition time  = 7 min, 27 sec); b) Functional MRI sequence for resting state: T2*-sensitive gradient echo spiral pulse sequence [28] (TR/TE: 2000/30 ms; 77° flip angle; Field of View (FOV)  = 22 cm, matrix  = 64×64) (results not described here); c) The functional scans were acquired using gradient-echo echo-planar imaging with the following parameters: (TR = 2s, TE = 27 milliseconds, FOV = 24 cm, acquisition matrix = 64×64, flip angle = 70°, voxel size = 3.75×3.75×4 millimeters, slice thickness = 4 millimeters, gap = 1 millimeter, 29 slices, ascending acquisition) [28].

#### Structural data acquisition

Volumetric segmentation was performed with the Freesurfer v. 5.1 image analysis suite, which is documented and freely available for download online (http://surfer.nmr.mgh.harvard.edu/). This processing was used to generate Estimated Total Intracranial Volume (eTIV) used for normalization. The technical details of these procedures are described in prior publications [29].

#### Task-related image processing and statistical analysis

Image processing was performed using Statistical Parametric Mapping (SPM 8: Wellcome Department of Cognitive Neurology, London, UK). The first 4 volumes of each functional series were discarded to allow T1 equilibrium. All functional images were then corrected for pilot motion and physiological noise. Each pilot's structural images were co-registered to the mean functional image and segmented into grey matter, white matter and cerebrospinal fluid. Skull stripping was done using Brain Xtract tool in SPM99 on T1 images. The grey matter volume was normalized to the Montreal Neurological Institute (MNI) grey matter template, and the normalization parameters were applied to all the structural and functional volumes. Functional images were spatially smoothed using a 8-mm Full width at half maximum (FWHM) Gaussian kernel.

Statistical analysis was performed using the general linear model implemented in SPM8. Each approach trial is 30–32 second long and a decision is made once during this time (at the DH of 200 ft, corresponding to approximately 18–22s). Therefore, an event related design based on the decision made was used. Specifically, the entire scan was modeled where pilot-specific regressors of interest were assembled by convolving delta functions (corresponding to the 2s prior to each decision) with a canonical hemodynamic response function (HRF). The control condition was modeled as baseline. Parameter estimates were used to calculate the appropriate linear contrasts. Movement regressors were also added.

We employed both functional (i.e., Neurosynth) and anatomical (i.e., Wake Forest University (WFU)Pick Atlas) criteria to generate specific ROI masks related to our hypothesis. First, we used a term-based, forward inference query to obtain a whole brain map of voxels related to decision-making (we used “decision-making” as the search term). This meta-analytic functional map was thresholded using cluster correction with an initial cutoff of z>4.5 and a corrected *p*<.05. Second, a visual inspection of the functional map was done using MRIcro software to identify the anatomical locations, mainly our ROI, bilateral caudate nucleus. Additional regions identified for secondary ROI analysis were bilateral insula, anterior cingulate, mid frontal and superior frontal regions. Third, WFUPick Atlas was used to create probabilistic anatomical masks of these regions. Finally, the Neurosynth functional map was constrained by the WFUPick Atlas anatomical masks using the ImCalc function in SPM8 (AND operator). The resulting maps used as ROI masks were more constrained and accurate as they were defined by both functional and anatomical criteria.

**Figure 2:**
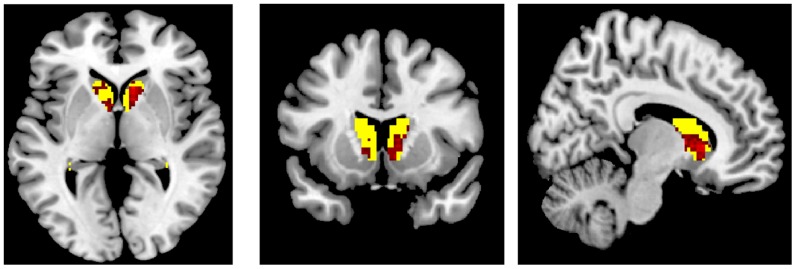
Primary Region of Interest (shown in red). Bilateral caudate anatomical mask derived from WFUPickAtlas (yellow) was overlaid with the combined ImCalc mask derived from Neurosynth (functional activity) and WFUPickAtlas (anatomical) in SPM 8 (shown in red). Axial, coronal and sagittal views shown.

We generated our primary ROI mask for bilateral caudate (see Figure 2) and additionally for insula, anterior cingulate, mid frontal and superior frontal regions. Marsbar toolbox in SPM 8 was used to extract task-related activation for: 1) All decisions (correct and incorrect decisions made in both “land” and “do not land” conditions); 2) Correct decisions (“land” and “do not land” conditions); and 3) Incorrect decisions (“land” and “do not land” conditions). Activity in each condition used a voxel-wise threshold of *p*<.01 uncorrected, minimum cluster size of 5 voxels for the primary and secondary ROIs (small volume correction was applied). These values were then exported to SAS for between-pilot and within-task condition analyses. Note: one High Expertise pilot decided to land every time and had no responses in the do not land condition, so the fMRI analysis included data from 19 pilots only.

## Results

### Demographics

Table 1 summarizes characteristics of the pilots at entry, grouped by expertise level. In terms of expertise, there were differences between the two groups in flight experience and age. As expected, more hours of total flight experience, overall *t* (12)  = 2.61, *p*<.05 and more hours of flight experience in the past month, *t* (11)  = 2.62, *p*<.001 were associated with higher expertise. In addition level of expertise was associated with younger age, *t* (18)  = −2.25, *p* = 0.045). There were no significant differences between the expertise groups in the remaining demographic measures (see Table 1 for means and SDs).

### Expertise Differences in Accuracy During the Landing Decision Task

High Expertise pilots were more accurate than Moderate Expertise pilots in making a “land” versus “do not land” decision (CFII/ATP: *d*′ = 3.62±2.52; IFR: *d*′ = 0.98±1.04; (t (18)  = 3.23; *p*<.01; ES = 1.37). The mean overall accuracy rate for the landing decision task was 71.43% (SD = 4.83%), indicating that pilots' decisions were above chance yet not at ceiling. As with *d*′ there was a significant difference in overall accuracy in the landing decision task between High Expertise pilots (CFII/ATP: Mean  = 80.44%±5.65%) and Moderate Expertise pilots (IFR: Mean  = 62.41%±4.01%) (t (18)  = 2.35, *p*<.05). As shown in Table 2, Moderate Expertise pilots made significantly more false alarms (i.e., more risky decisions to descend below the minimum safe altitude) than High Expertise pilots; (t (18)  = −2.32; *p*<.05).

**Table 2:** Responses made during Landing Decision Task by Expertise: proportion of hits, misses, false alarms, correct rejections and *d*′ (High Expertise  =  CFII/ATP; Moderate Expertise  =  IFR).

Expertise	N	Variable	N	Mean	Std Dev	Minimum	Maximum
CFII/ATP	12	hit_prop	12	0.82	0.28	0.11	1
		miss_prop	12	0.18	0.28	0	0.89
		false_alarm_prop	12	0.21	0.31	0	1
		corr_reject_prop	12	0.79	0.31	0	1
		d_prime	12	3.62	2.52	−0.55	7.26
							
IFR	8	hit_prop	8	0.77	0.16	0.61	1
		miss_prop	8	0.23	0.16	0	0.39
		false_alarm_prop	8	0.52	0.28	0.08	0.94
		corr_reject_prop	8	0.48	0.28	0.06	0.92
		d_prime	8	0.98	1.04	−0.37	2.70

### Expertise Differences in Brain Activation During the Landing Decision Task

#### Main hypothesis

**Figure 3:**
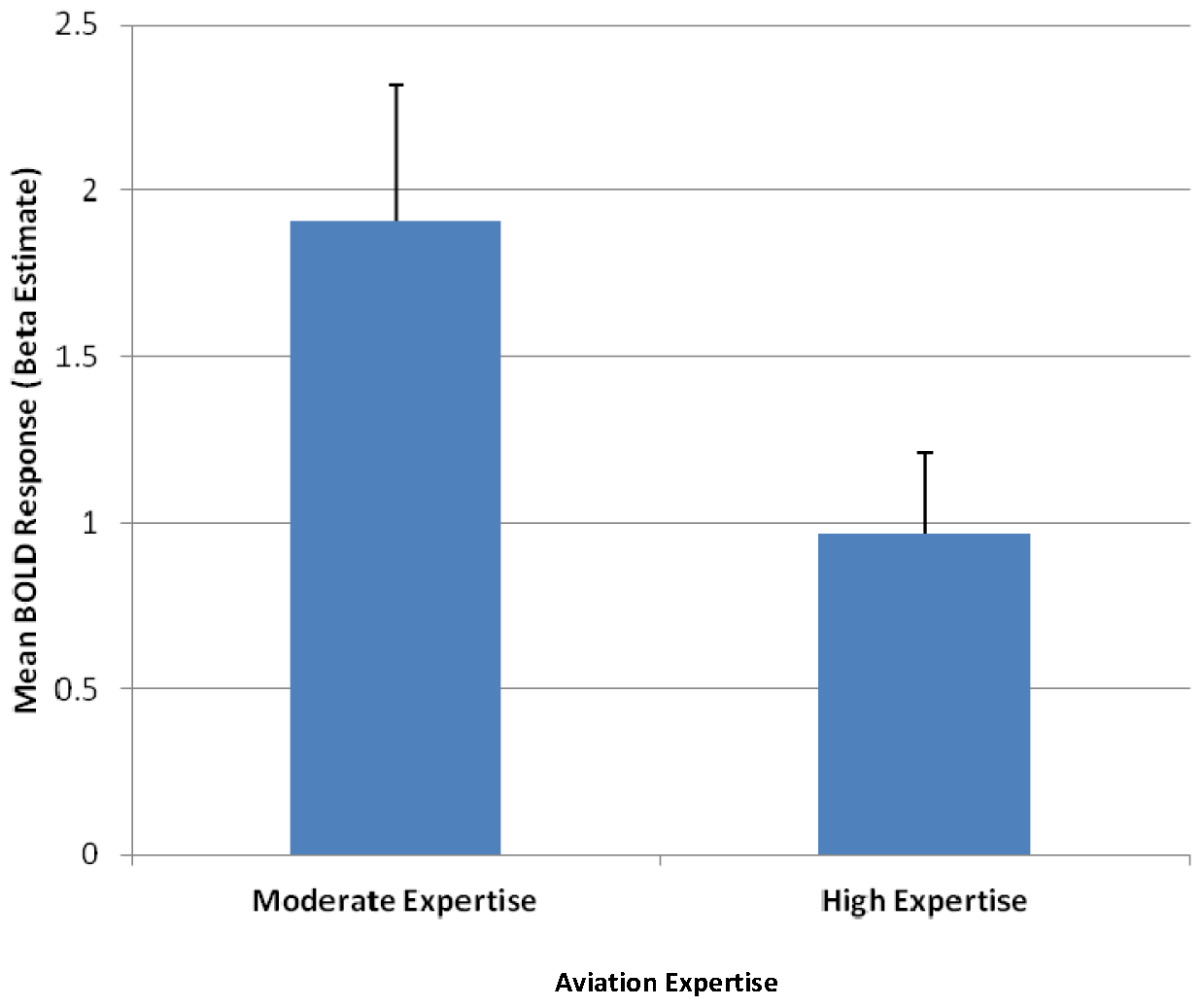
Activity During all Decisions in Primary Region of Interest. The mean estimated blood oxygen level dependent (BOLD) response for all decisions in bilateral caudate region during the landing decision task by expertise. High expertise  =  CFII/ATP; Moderate expertise  =  IFR. Error bars designate standard error.

We focused on bilateral caudate region in our main hypothesis to investigate the role of expertise and skill learning in a real-world decision-making task. Lower brain activation was observed during all decisions made (correct and incorrect decisions from land and do not land conditions) in High Expertise pilots (0.97±0.80) compared to Moderate Expertise pilots (1.91±1.16) in bilateral caudate nucleus (t (17)  = 2.1, *p* = .0497; ES = 0.46; see Figure 3).

#### Post hoc analyses

We looked at bilateral caudate activity during correct and incorrect decisions separately. The differences in mean activation between Moderate and High Expertise were not significant but were in the same direction as the main analysis. In addition, we also examined *d*', a measure of sensitivity to the runway environment, in relation to bilateral caudate activity. D prime was specifically and negatively correlated with activity in the bilateral caudate region during correct decisions (r = −0.61, *p*<.01), but not during incorrect or even all decisions.

#### Secondary ROIs

We also extracted activation from bilateral insula, anterior cingulate, middle frontal and superior frontal regions for all decisions made. Although there were no statistically significant expertise-related differences in these secondary ROIs, the bilateral middle frontal region approached significance (*p* = .051). Activity tended to be lower in High Expertise pilots (0.61±0.50) than in Moderate Expertise pilots (1.29±0.88). During incorrect decisions, activity in bilateral superior frontal region was lower for High Expertise pilots (0.51±0.50) compared to Moderate Expertise pilots (2.26±1.43) (*p* = .0174).

### Age Differences in Accuracy During the Landing Decision Task

Because the two expertise groups differed in age, age differences in performance and brain activity were explored. In terms of landing decision accuracy, the effect of age on *d*′ was not significant (older: *d*′ = 1.79±2.45; younger: *d*′ = 3.33±2.23; *p*>.1). Age differences in flight control during the approach maneuver were observed. Older pilots also made larger joystick (aileron) movements, indicating more roll of the aircraft along its longitudinal axis (*p* = .013). Thus, older age was associated with less precise control of the aircraft during the simulated approach. (Note: expertise was not significantly associated with any measure of flight control during the approach; *p*'*s*>.1).

### Age Differences in Brain Activation During the Landing Decision Task

Age differences in BOLD response during all decisions made were not significant for the primary ROI, bilateral caudate, (*p* = .965). During correct decisions, pilots over the age of 45 showed a nonsignificant trend for greater activity in the bilateral caudate compared to younger pilots (*p* = .058; ES = 0.39). The relationship between age and bilateral caudate activity was such that older pilots had greater activity (*r* = 0.6) compared to younger pilots.

#### Secondary ROIs

In making all decisions younger pilots showed a trend for greater activity in the bilateral insula (1.36±0.86) compared to older pilots (0.67±0.49; t (17)  = −2.17, *p* = .050). Age-related differences were not observed in any other brain regions or decision conditions.

## Discussion

The present study provides evidence regarding the role of the caudate in making the crucial decision to land in poor-visibility weather. First, we found that High Expertise pilots were more accurate than Moderate Expertise pilots in making a “land” versus “do not land” decision during a simulated heavy fog (legally unsafe to land) and less dense fog (legally safe to land) scenario. Second, the expertise effect on landing-decision accuracy was simultaneously reflected in lower activity in our primary region of interest, bilateral caudate. Specifically, High Expertise pilots had lower activation in bilateral caudate with fewer erroneous decisions. In contrast, Moderate Expertise pilots had greater activation in bilateral caudate even though their decisions were less accurate. Overall, an expertise effect of higher landing-decision accuracy was observed, accompanied by lower activity in bilateral caudate region. The expert aviation knowledge required to make a decision relevant to safety in a real-world outcome adds a novel perspective to the extensive research conducted on decision making and its neural basis.

The main body of decision making research focuses on decision making with financial incentives, and is based on the Expected Value (EV) theory, which states that the decision maker chooses the option that has the most EV while weighing the probabilities related to the payoffs [30]. Experimental tasks involving financial risk-taking ask participants to decide among choices in which the odds explicitly favor one of the available options, rewarding or punishing choices with arbitrary points. In an aviation decision-making study, Causse et al., [4] adopted a simplified landing-decision task to test a similar economic hypothesis where financial reward and the certainty of landing safely were manipulated. When there was a financial incentive and high uncertainty, pilots made more risky decisions, as measured in terms of greater willingness to land despite uncertainty. In addition, pilots made faster decisions when financial incentives were offered; and when uncertainty of the outcome was high, heart rate (HR) increased, heart rate variability (HRV) decreased, and pilots' eyes tracking data showed longer fixations on the ILS instrument. In the current fMRI compatible landing decision task no financial incentives were given for a correct decision. Mirroring a real-world scenario, the landing decision task was designed to rely on the pilot's motivation to make a safe landing according the FAA rules based on DH.

Our results show lower functional brain activity in pilots with high aviation expertise during decision making. This lower activity in experts could be associated with a lower amount of saccades or a lower attentional effort and a better “neural efficiency” in general. Regarding saccades, it is important to note that this decision was aided by a pilot's eye movements during an ILS approach that are directed at the instruments and at landing visual markers at the DH. As stated in the FAA Instrument Flying Handbook, “With increasing experience… a pilot learns what to look for, [and] when to look for it. (Chapter 4, p. 11).” For instance, Kasarskis et al. [31] found that during a visual approach and descent, the expert pilots had more fixations and shorter dwell times than novice pilots. However, as they approached the runway, where experts and novices looked differed. Experts had a more “clearly defined pattern” of saccades transitioning back and forth between the runway aimpoint and the airspeed instrument. In contrast, novices had a less defined, “more complicated pattern” of saccades as they approach the runway. For example, one novice had several instances of horizontal eye movements within the runway area itself and fixated on both the airspeed and altimeter instruments. Basal ganglia, as mentioned earlier, is a key player in controlling small voluntary motor movement and one of the most intensively studied structures in eye movement research [20]. Our results are in line with the idea that the basal ganglia, particularly the caudate nucleus, is a key region involved in the mechanism by which eye-motor saccades play a role in correct and safe landing decisions. When sensory information is absent or no new relevant information is available, the basal ganglia come on-line. By contrast, when relevant information is visually available, the basal ganglia activation may be lower [20].

The lower activity in bilateral caudate region in experts during decision making could also reflect overall lower attentional effort/better “neural efficiency” more generally. For instance, Peres et al [6] observed relatively localized brain activation in experts (fighter or military transport pilots) during a simulated aviation track-following task, in contrast to novices (student pilots) who showed widespread brain activation in regions that included prefrontal, parietal, temporal, visual and motor/somatosensory cortices. A very recent study investigated the expertise related functional brain differences in Formula one race-car drivers compared to ‘naïve’ drivers [32]. Although performance was similar in both groups during the motor reaction and visuo-spatial tasks, lower task-related activity was observed in the professional race car drivers. These results point to increased ‘neural efficiency’ among expert, high-performance pilots and drivers. Other fMRI studies of expert performance reveal more activation of task-dependent brain regions in experts than novices; for instance, basketball players (bilateral inferior frontal gyrus and right anterior insular cortex; [33]), badminton players (medial, dorsolateral and ventrolateral frontal cortex; [34]), opera singers (basal ganglia, the thalamus, and the cerebellum; [35]) and archers (middle frontal cortex, supplemental motor area, and dorsolateral prefrontal cortex; [36]). A recent fMRI study relevant to our results compared professional Shogi (Japanese chess) players with amateurs. Based on the performance, the quick generation of the best next-move occurred more reliably in professionals than in amateurs, and this was associated with stronger activation in the head of caudate nucleus, a dorsal part of the basal ganglia [37]. These and related findings [38] led these researchers to indicate the caudate nucleus as the region involved in automating cognitive computation that leads to the “best next-move” [38]. Taken together, our results are generally consistent with studies of expert pilots and drivers, which provide evidence for “neural efficiency” of the brain regions relevant to the performance of particular task or specialized skill.

Our primary aim in this study was to characterize, in terms of performance and brain activity, the contribution of aviation expertise to the crucial decisions pilots make when they land in bad weather. In terms of performance, our results show that higher expertise is associated with more accurate decision-making in a simulator-based landing decision task inside an fMRI scanner. These results are consistent with previous results on expertise and performance of aviation tasks [39], [31], [13], [40]–[42]. These studies reported a variety of performance differences related to expertise level; however, these studies involved pilots with a wider range of expertise, including Visual Flight Rule (VFR) pilots as the least expert group of licensed pilots. (A VFR rating allows pilots to fly under visual flight rules only, which limits them to flying only in good weather conditions.) Given the inclusion/exclusion criteria of our study of IFR flying, VFR pilots were excluded from our study. It is noteworthy that, in the current study, significant expertise effects were present, even though expertise varied from high to moderate, and did not include low expertise participants, such as VFR and student pilots.

Similar to other novel fMRI studies, a limitation of this study is the relatively small number of participants, which resulted in a modest statistical power. As such, hypothesis testing was focused on a single ROI (bilateral caudate) and there was modest power to detect between-group differences, especially differences related to age. Age played a role in this study as pilots that were younger had better flight control than older pilots (*p* = .013) and High Expertise pilots were younger than Moderate Expertise pilots, on average (*p* = .045). Although age differences in bilateral caudate activity were not significant during all decisions made (*p* = .965), post hoc analyses revealed a nonsignificant trend for age differences in bilateral caudate activity during correct decisions (*p* = .058; ES = 0.39). In view of the younger age of High Expertise pilots compared to Moderate Expertise pilots, conclusions regarding increased “neural efficiency” could be related to younger age, higher expertise level, or both. The data and results of the present study are not rich enough to distinguish between these interpretations. In the literature, there are a number of reports on significant age (and expertise-related) differences in performance of aviation tasks [11]; [40], [43]. Our laboratory previously reported that only age and speed of processing explained decision making in hazardous conditions in civilian pilots tested in a flight simulator [13]. For these reasons, a larger sample in a future study is needed to tease apart the effects of age and expertise. Second, we focused on making a real-world simulated task compatible with an fMRI task design and it constrained us in some ways. Although the task design and *a priori* hypothesis focused on the caudate nucleus, other regions known to be involved in decision making would also provide additional information about expertise and age-related differences. If the balance between the real-world and experimental fMRI task design can be developed further, an Independent Component Analysis approach (ICA) [44] could be utilized to account for dynamic aspects of stimuli used in real-world decision making. Third, inclusion of novice pilots in a similar study would provide further insight into the difference between visual search training (at DH) and aviation expertise required to make the correct decision. Finally, we did attempt to collect eye tracking data initially in this study, but lighting conditions inside the bore of the magnet and other technical difficulties dissuaded us from collecting this data any further. Future studies, including those that are designed to better understand the saccade behavior during IFR landing approaches, will greatly enhance understanding of the mechanisms behind neural efficiency and attentional control.

Our findings have both methodological and practical implications. First, our real-world task design approach used inside the fMRI scanner demonstrates that a simulated real-world task can be used reliably to assess effects of expertise and skill on performance and brain activity simultaneously in a specialized population. Second, our study was further strengthened by the use of *Neurosynth* [45] –a method that allowed us to use functionally- and anatomically-derived primary region of interest in our study. Finally, real-world tasks can be used as a platform for deliberate practice and improved risk perception, where performance and brain activity can be targeted to improve decision making process especially in hazardous conditions. In fact, monitoring brain activity, such as in bilateral caudate, might prove to be an effective means for monitoring cognitive states and task engagement, which could be valuable for training recruits especially in military settings. Aviation safety researchers are currently developing cognitive-state monitoring systems using Functional Near Infrared Spectroscopy (fNIRS), fMRI and Electroencephalography (EEG) to distinguish between high and low levels of task engagement [46] and workload [5]. Once developed, these portable neuroimaging methods can be applied in real-world settings, for example, in simulator settings where pilots undergo training (e.g. simulated flight emergencies) or re-certification following medical treatment. Such practical applications are important, given that weather-related accidents have the highest rates of fatality (71%) among US general aviation accidents [7]. In fact, in approximately 2000 cases of approaches in severe weather conditions, two out of three commercial aircrews chose to land [47]. The ‘urge to land’ phenomenon (also known as the “get-home-itis syndrome” by pilots and “plan continuation error” by aviation psychologists [48] has been implicated in more than 41.5% of casualties in civil aviation (as reported by the French Accident Investigation Bureau [49], [8]. Given the fatal outcomes of such decisions, it is important to understand which factors affect decision making processes in hazardous conditions and ways in which these errors can be prevented. Our results have implications for better understanding cognitive processes associated with specialized training and its application in the real world, especially in safety.

## Supporting Information

Table S1 Raw Data for all Analyses Reported in the Manuscript.
(XLSX)

## References

[1] Stearns SC (2000) Daniel Bernoulli (1738): evolution and economics under risk. J. Biosci 25:221–8. 11022222 10.1007/BF02703928

[2] Huettel SA, Stowe CJ, Gordon EM, Warner BT, Platt ML (2006) Neural signatures of economic preferences for risk and ambiguity. Neuron 49:765–775. 16504951 10.1016/j.neuron.2006.01.024

[3] Schonberg T, Fox CR, Poldrack RA (2011) Mind the gap: bridging economic and naturalistic risk-taking with cognitive neuroscience. Trends Cogn Sci 15:11–19. 21130018 10.1016/j.tics.2010.10.002 PMC3014440

[4] Causse M, Baracat B, Pastor J, Dehais F (2011) Reward and uncertainty favor risky decision-making in pilots: evidence from cardiovascular and oculometric measurements. Appl Psychophysiol Biofeedback 36:231–242. 21739293 10.1007/s10484-011-9163-0

[5] Durantin G, Gagnon JF, Tremblay S, Dehais F (2014) Using near infrared spectroscopy and heart rate variability to detect mental overload. Behav Brain Res 259:16–23. 24184083 10.1016/j.bbr.2013.10.042

[6] Peres M, Van De Moortele PF, Pierard C, Lehericy S, Satabin, et al (2000) Functional magnetic resonance imaging of mental strategy in a simulated aviation performance task. Aviat Space Environ Med 71:1218–1231. 11439722

[7] AOPA (2007) Nall Report: Accident Trends and Factors for 2006. Frederick MD: AOPA Air Safety Foundation.

[8] Causse M, Peran P, Dehais F, Caravasso CF, Zeffiro T, et al (2013) Affective decision making under uncertainty during a plausible aviation task: an fMRI study. Neuroimage 71:19–29. 23313780 10.1016/j.neuroimage.2012.12.060

[9] Adamson MM, Samarina V, Xiangyan X, Huynh V, Kennedy Q, et al (2010) The impact of brain size on pilot performance varies with aviation training and years of education. J Int Neuropsychol Soc 16:412–423. 20193103 10.1017/S1355617710000111 PMC2862858

[10] Adamson MM, Bayley PJ, Scanlon BK, Farrell ME, Hernandez B, et al (2012) Pilot expertise and hippocampal size: associations with longitudinal flight simulator performance. Aviat Space Environ Med 83:850–857. 22946348 10.3357/asem.3215.2012 PMC7199657

[11] Taylor JL, Kennedy Q, Noda A, Yesavage JA (2007) Pilot age and expertise predict flight simulator performance: a 3-year longitudinal study. Neurology 68:648–654. 17325270 10.1212/01.wnl.0000255943.10045.c0 PMC2907140

[12] Yesavage JA, Jo B, Adamson MM, Kennedy Q, Noda A, et al (2011) Initial cognitive performance predicts longitudinal aviator performance. J Gerontol B Psychol Sci Soc Sci 66:444–453. 21586627 10.1093/geronb/gbr031 PMC3132267

[13] Kennedy Q, Taylor JL, Reade G, Yesavage JA (2010) Age and expertise effects in aviation decision making and flight control in a flight simulator. Aviat Space Environ Med 81:489–497. 20464816 10.3357/asem.2684.2010 PMC2905035

[14] Bechara A, Damasio AR, Damasio H, Anderson SW (1994) Insensitivity to future consequences following damage to human prefrontal cortex. Cognition 50:7–15. 8039375 10.1016/0010-0277(94)90018-3

[15] Lejuez CW, Read JP, Kahler CW, Richards JB, Ramsey SE, et al (2002) Evaluation of a behavioral measure of risk taking: the Balloon Analogue Risk Task (BART). J Exp Psychol Appl 8:75–84. 12075692 10.1037//1076-898x.8.2.75

[16] Ernst M, Paulus MP (2005) Neurobiology of decision making: a selective review from a neurocognitive and clinical perspective. Biol Psychiatry 58:597–604. 16095567 10.1016/j.biopsych.2005.06.004

[17] Arce E, Santisteban C (2006) Impulsivity: a review. Psicothema 18:213–220. 17296034

[18] Goudriaan AE, Oosterlaan J, de Beurs E, Van den Brink W (2004) Pathological gambling: a comprehensive review of biobehavioral findings. Neurosci Biobehav Rev 28:123–141. 15172761 10.1016/j.neubiorev.2004.03.001

[19] Tunik E, Houk JC, Grafton ST (2009) Basal ganglia contribution to the initiation of corrective submovements. Neuroimage 47:1757–1766. 19422921 10.1016/j.neuroimage.2009.04.077 PMC6368854

[20] Shires J, Joshi S, Basso MA (2010) Shedding new light on the role of the basal ganglia-superior colliculus pathway in eye movements. Curr Opin Neurobiol 20:717–725. 20829033 10.1016/j.conb.2010.08.008 PMC3008502

[21] DiChiara G, Porceddu ML, Morelli M, Mulas ML, Gessa GL (1979) Evidence for a GABAergic projection from the substantia nigra to the ventromedial thalamus and to the superior colliculus of the rat. Brain Res 176:273–284. 40667 10.1016/0006-8993(79)90983-1

[22] Mann DL, Spratford W, Abernethy B (2013) The head tracks and gaze predicts: how the world's best batters hit a ball. PLoS One 8:e58289. 23516460 10.1371/journal.pone.0058289 PMC3596397

[23] Crespi S, Robino C, Silva O, de'Sperati C (2012) Spotting expertise in the eyes: billiards knowledge as revealed by gaze shifts in a dynamic visual prediction task. J Vis 12:11. 10.1167/12.11.30 23115218

[24] Yang JH, Kennedy Q, Sullivan J, Fricker RD (2013) Pilot performance: assessing how scan patterns & navigational assessments vary by flight expertise. Aviat Space Environ Med 84:116–124. 23447849 10.3357/asem.3372.2013

[25] Poldrack RA, Kittur A, Kalar D, Miller E, Seppa C, et al (2011) The cognitive atlas: toward a knowledge foundation for cognitive neuroscience. Front Neuroinform 5:17. 21922006 10.3389/fninf.2011.00017 PMC3167196

[26] Yarkoni T, Poldrack RA, Nichols TE, Van Essen DC, Wager TD (2011) Large-scale automated synthesis of human functional neuroimaging data. Nat Methods 8:665–670. 21706013 10.1038/nmeth.1635 PMC3146590

[27] Poldrack RA (2006) Can cognitive processes be inferred from neuroimaging data? Trends Cogn Sci 10:59–63. 16406760 10.1016/j.tics.2005.12.004

[28] Glover GH, Lai S (1998) Self-navigated spiral fMRI: interleaved versus single-shot. Magn Reson Med 39:361–368. 9498591 10.1002/mrm.1910390305

[29] Fischl B (2012) FreeSurfer. Neuroimage 62:774–781. 22248573 10.1016/j.neuroimage.2012.01.021 PMC3685476

[30] Rao LL, Li S, Jiang T, Zhou Y (2012) Is payoff necessarily weighted by probability when making a risky choice? Evidence from functional connectivity analysis. PLoS One 7:e41048. 22815908 10.1371/journal.pone.0041048 PMC3398869

[31] Kasarskis P, Stehwien J, Hickox J, Aretz A (2001) Comparison of expert and novice scan behaviors during VFR flight. Presented at the 11^th^ International Symposium on Aviation Psychology. Columbus, OH.

[32] Bernardi G, Ricciardi E, Sani L, Gaglianese A, Papasogli A, et al (2013) How skill expertise shapes the brain functional architecture: an fMRI study of visuo-spatial and motor processing in professional racing-car and naive drivers. PLoS One 8:e77764. 24204955 10.1371/journal.pone.0077764 PMC3799613

[33] Abreu AM, Macaluso E, Azevedo RT, Cesari P, Urgesi C, et al (2012) Action anticipation beyond the action observation network: a functional magnetic resonance imaging study in expert basketball players. Eur J Neurosci 35:1646–1654. 22541026 10.1111/j.1460-9568.2012.08104.x

[34] Wright MJ, Bishop DT, Jackson RC, Abernethy B (2010) Functional MRI reveals expert-novice differences during sport-related anticipation. Neuroreport 21:94–98. 20051784 10.1097/WNR.0b013e328333dff2

[35] Kleber B, Veit R, Birbaumer N, Gruzelier J, Lotze M (2009) The brain of opera singers: experience-dependent changes in functional activation. Cereb Cortex 20:1144–1152. 19692631 10.1093/cercor/bhp177

[36] Seo J, Kim YT, Song HJ, Lee HJ, Lee J, et al (2012) Stronger activation and deactivation in archery experts for differential cognitive strategy in visuospatial working memory processing. Behav Brain Res 229:185–193. 22266924 10.1016/j.bbr.2012.01.019

[37] Wan X, Nakatani H, Ueno K, Asamizuya T, Cheng K, et al (2011) The neural basis of intuitive best next-move generation in board game experts. Science 331:341–346. 21252348 10.1126/science.1194732

[38] Wan X, Takano D, Asamizuya T, Suzuki C, Ueno K, et al (2012) Developing intuition: neural correlates of cognitive-skill learning in caudate nucleus. J Neurosci 32:17492–17501. 23197739 10.1523/JNEUROSCI.2312-12.2012 PMC6621838

[39] Bellenkes AH, Wickens CD, Kramer AF (1997) Visual scanning and pilot expertise: The role of attentional flexibility and mental model development. Aviation Space and Environmental Medicine 68:569–579. 9215461

[40] Morrow DG, Miller LM, Ridolfo HE, Menard W, Stine-Morrow EA, et al (2005) Environmental support for older and younger pilots' comprehension of air traffic control information. J Gerontol B Psychol Sci Soc Sci 60:P11–18. 15643033 10.1093/geronb/60.1.p11

[41] Schriver AT, Morrow DG, Wickens CD, Talleur DA (2008) Expertise differences in attentional strategies related to pilot decision making. Human Factors 50:864–878. 19292010 10.1518/001872008X374974

[42] Taylor JL, O'Hara R, Mumenthaler MS, Rosen AC, Yesavage JA (2005) Cognitive ability, expertise, and age differences in following air-traffic control instructions. Psychol Aging 20:117–133. 15769218 10.1037/0882-7974.20.1.117

[43] Van Benthem K, Herdman CM, LeFevre J (2011) Prospective Memory Tasks in Aviation: Effects of Age and Working Memory. Accepted for presentation at the 32nd Annual Conference of the Cognitive Science Society.

[44] Calhoun VD, Adalı T, Pearlson GD, van Zijl PCM, Pekar JJ (2002) Independent component analysis of fMRI data in the complex domain. Magn Reson Med 48:180–192. 12111945 10.1002/mrm.10202

[45] Chang LJ, Yarkoni T, Khaw MW, Sanfey AG (2012) Decoding the role of the insula in human cognition: functional parcellation and large-scale reverse inference. Cereb Cortex 23:739–749. 22437053 10.1093/cercor/bhs065 PMC3563343

[46] Harrivel AR, Weissman DH, Noll DC, Peltier SJ (2013) Monitoring attentional state with fNIRS. Front Hum Neurosci 13:861. 10.3389/fnhum.2013.00861 PMC3861695 24379771

[47] Rhoda D, Pawlak M (1999) An Assessment of Thunderstorm Penetrations and Deviations by Commercial Aircraft in the Terminal Area. Massachusetts Institute of Technology, Lincoln Laboratory.

[48] Orasanu J, Ames N, Martin L, Davison J (2001) Factors in aviation accidents: decision errors. Mahwah, NJ: Lawrence Erlbaum Associates.

[49] BEA (2000) Objectif: Destination: Technical Report Bureau Enquête Analyse.

